# Therapeutic mechanism of Toujie Quwen granules in COVID-19 based on network pharmacology

**DOI:** 10.1186/s13040-020-00225-8

**Published:** 2020-09-24

**Authors:** Ying Huang, Wen-jiang Zheng, Yong-shi Ni, Mian-sha Li, Jian-kun Chen, Xiao-hong Liu, Xing-hua Tan, Ji-qiang Li

**Affiliations:** 1 First College of Clinical Medicine, Guangzhou University of Chinese Medicine, Guangzhou, China; 2 Integrative Dept.3 (Geriatrics Dept), Guangdong Provincial Hospital of Chinese Medicine, Guangzhou, China; 3 The Second College of Clinical Medicine, Guangzhou University of Chinese Medicine, Guangzhou, China; 4 Tianhe Shadong Street Community Healthcare Service Center, Guangzhou, China; 5 Department of Traditional Chinese Medicine, Guangzhou Eighth People’s Hospital, Guangzhou, China

**Keywords:** COVID-19, Toujie Quwen granule, Potential therapeutic targets, Signaling pathways, Network pharmacology

## Abstract

**Background:**

Chinese medicine Toujie Quwen granule (TJQW) has proven to be effective in the treatment of mild coronavirus disease 2019 (COVID-19) cases by relieving symptoms, slowing the progression of the disease, and boosting the recovery of patients. But the bioactive compounds and potential mechanisms of TJQW for COVID-19 prevention and treatment are unclear. This study aimed to explore the potential therapeutic mechanism of TJQW in coronavirus disease 2019 (COVID-19) based on an integrated network pharmacology approach.

**Methods:**

TCMSP were used to search and screen the active ingredients in TJQW. The Swiss TargetPrediction was used to predict the potential targets of active ingredients. Genes co-expressed with ACE2 were considered potential therapeutic targets on COVID-19. Venn diagram was created to show correlative targets of TJQW against COVID-19. Cytoscape was used to construct a “drug-active ingredient-potential target” network, STRING were used to construct protein-protein interaction network, and cytoHubba performed network topology analysis. Enrichment of biological functions and signaling pathways of core targets was performed by using the clusterProfiler package in R software and ClueGO with CluePedia plugins in Cytoscape.

**Results:**

A total of 156 active ingredients were obtained through oral bioavailability and drug-likeness screenings. Two hundred twenty-seven potential targets of TJQW were related to COVID-19. The top ten core targets are EGFR, CASP3, STAT3, ESR1, FPR2, F2, BCL2L1, BDKRB2, MPO, and ACE. Based on that, we obtained 19 key active ingredients: umbelliprenin, quercetin, kaempferol, luteolin, praeruptorin E, stigmasterol, and oroxylin A. And the enrichment analysis obtained multiple related gene ontology functions and signaling pathways. Lastly, we constructed a key network of “drug-component-target-biological process-signaling pathway”. Our findings suggested that TJQW treatment for COVID-19 was associated with elevation of immunity and suppression of inflammatory stress, including regulation of inflammatory response, viral process, neutrophil mediated immunity, PI3K-Akt signaling pathway, MAPK signaling pathway, Jak-STAT signaling pathway, Complement and coagulation cascades, and HIF-1 signaling pathway.

**Conclusions:**

Our study uncovered the pharmacological mechanism underlying TJQW treatment for COVID-19. These results should benefit efforts for people around the world to gain more knowledge about Chinese medicine TJQW in the treatment of this vicious epidemic COVID-19, and help to address this pressing problem currently facing the world.

## Introduction

Since December 2019, a novel coronavirus pneumonia, namely coronavirus disease 2019 (COVID-19) has rapidly spread from Wuhan City to various provinces across China and other countries around the world [1]. Update to 29 July 2020, there were 16,341,920 confirmed cases, 650,805 confirmed deaths, and 216 countries, areas or territories with cases, according to the WHO. (https://www.who.int/emergencies/diseases/novel-coronavirus-2019). As an acute respiratory infectious disease, COVID-19 is transmitted through respiratory droplets and close contact, and the general population is susceptible to the infection. No specific drug but symptomatic supporting treatment is currently available for COVID-19. According to the traditional Chinese medicine (TCM) theory, COVID-19 belongs to the category of pestilence. Since the outbreak of COVID-19, the Chinese government has explicitly requested adherence to the integration of TCM and western medicine, enhancement of the research efforts, and acceleration of the development of drugs with good clinical efficacy for COVID-19. The Diagnosis and Treatment Program of Novel Coronavirus Pneumonia (trial editions 4 to 7) jointly issued by the National Health Commission of China and the National Administration of TCM of the People’s Republic also included a TCM treatment section [2].

Among all recommended TCM formulations for COVID-19 treatment, Guangzhou Eighth People’s Hospital (http://www.gz8h.com.cn/, Guangdong Province, China) introduced Toujie Quwen granules (TJQW, formerly known as pneumonia No. 1 formulation) based on the febrile disease theory of TCM and climate characteristics of Lingnan region, TJWQ formulation has achieved good therapeutic results. TJQW have been mainly used to treat mild to moderate COVID-19 cases to effectively reduce fever, cough, and expectoration in the patients. On February 8, 2020, Guangdong Medical Products Administration officially recommended TJQW as COVID-19 treatment to 30 designated hospitals for COVID-19 in the Guangdong Province [3]. TJQW includes 16 TCM components, including *Forsythiae Fructus* (lian-qiao), edible tulip (shan-ci-gu), *Lonicera japonica* (Japanese honeysuckle flower, jin-yin-hua), *Radix Scutellariae baicalensis* (huang-qin), *Folium Isatidis* (da-qing-ye), *Bupleurum* root (chai-hu), *Artemisia apiacea* (qing-hao), *Periostracum Cicadae* (chan-tui), *Radix Peucedani* (qian-hu), *Fritillaria cirrhosa* (chuan-bei-mu), *Fritillaria thunbergii* (zhe-bei-mu), *Poria cocos* (fu-ling), *Fructus Mume* (wu-mei), radix *Scrophulariae* (xuan-shen), *Astragalus propinquus* (huang-qi), and radix *Pseudostellariae* (tai-zi-shen), which were mainly used for heat-clearing, detoxifying, relieving symptoms, expelling pathogenic factors, reinforcing *qi*, and nourishing *yin* according to the TCM theory [4].

TCM and Western medicine may come from two different medical systems and have different perspectives on health and diseases, but they are both based on the standard of factual clinical efficacy. Network pharmacology has emerged as a powerful tool to reveal active ingredients, biological targets and signaling pathways linked to certain diseases [5, 6]. The therapeutic target for COVID-19 remains unclear, while study has shown that the novel coronavirus infects alveolar type II cells through the mechanism of interaction of the spike protein with the human renin and type II angiotensin converting enzyme (ACE2), leading to pneumonia [7], therefore, genes co-expressed with ACE2 are considered as potential therapeutic targets on COVID-19 [8]. ACE2 is expressed by epithelial cells of the lungs at a high level, a major target of the disease, SARS-CoV-2 infects ACE2-expressing cells in the lung, blocking ACE2 interaction with the S protein of SARS-CoV-2 to curtail SARS-CoV-2 infection are becoming very attractive therapeutics potential for treatment and prevention of COVID-19, thus, we could target this interaction site with small molecules [9]. We analyzed the co-expressed genes with ACE2 in order to explore different target genes on which the small molecules could affect the SARS-CoV-2 activity [10].

**Fig. 1:**
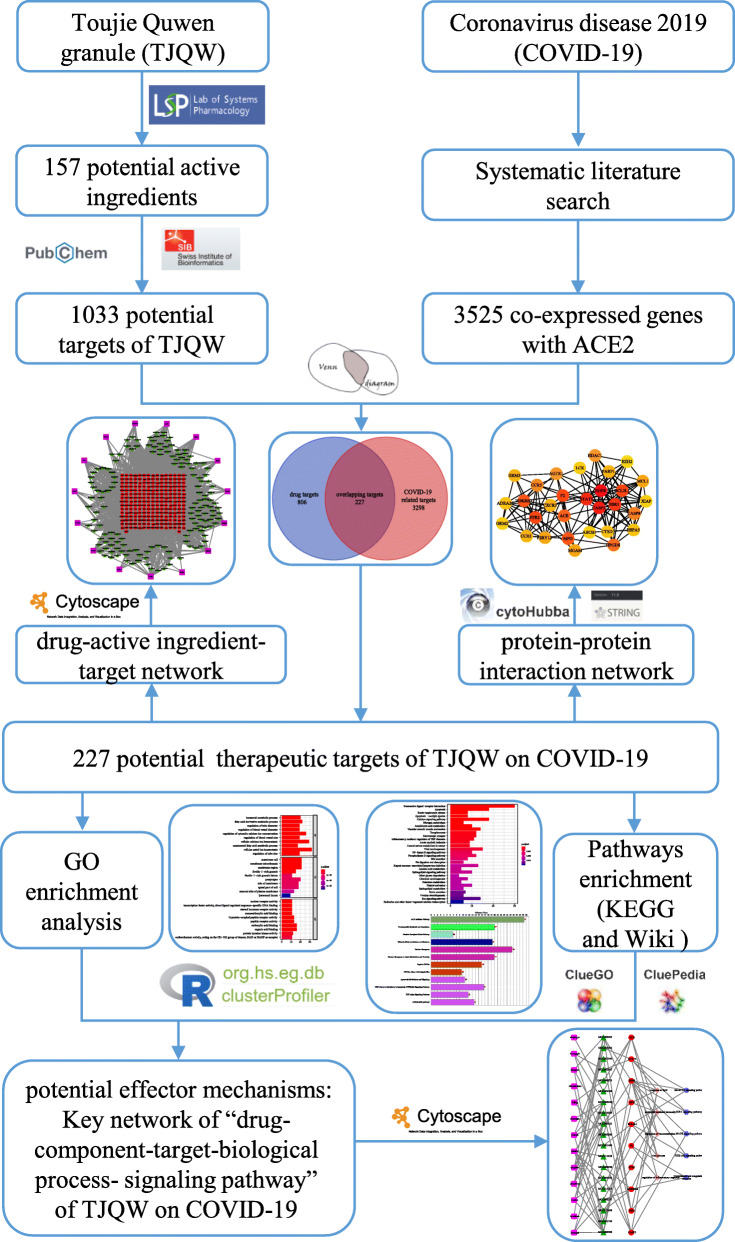
Overall workflow of this study

This study selected the active ingredients of TJQW and used the network pharmacology approach to construct a common target for the drug and COVID-19, to further explore the effective ingredients and molecular mechanisms of this TCM formulation for COVID-19, and to provide further scientific basis for the study of TCM treatments for COVID-19, the overall workflow of this study was presented in Fig. 1.

## Materials and methods

### Screening of potential biologically active ingredients in TJQW

TJQW had a total of 16 TCM components, and the active ingredients in the related TCM components were searched through the TCM systems pharmacology database and the analysis platform (LSP, https://tcmspw.com/tcmsp.php) [11]. LSP captures relationships between drugs, targets, and diseases, includes pharmacokinetic properties for natural compounds involving oral bioavailability (OB), drug-likeness (DL), and this has started a trend in the search for candidate drugs in various types of traditional Chinese herbs. The TCM formulae must first overcome the barriers posed by absorption, distribution, metabolism, and excretion (ADME) processes, and only the molecules that pass through those barriers may be active. High OB is usually a key indicator for determining the DL index of bioactive molecules [12, 13]. Therefore, in this study, we screened bioactive molecules according to the pharmacokinetic parameters of OB ≥30% and DL ≥0.18 screening conditions. Then we obtained the chemical abstracts service (CAS) number or the IUPAC International Chemical Identifier (InChIKey) of those molecules from TCMSP.

### Prediction of potential targets for bioactive ingredients

The active ingredients obtained from screening were entered into the PubChem (https://pubchem.ncbi.nlm.nih.gov/) using their CAS number or InChIKey to acquire the corresponding molecular structures which were stored in canonical simplified molecular-input line-entry system (SMILES) format.

The Swiss TargetPrediction (http://www.swisstargetprediction.ch/, version 2019) [14] was then used to predict the potential targets of the active ingredients. We input the aforementioned potential active ingredients in SMILES format into this database, with “humans” (*Homo sapiens*) as the study species, to obtain the potential effector targets.

### Selection of the intersection of the drug targets and disease targets

Our study was based on the results of single-cell sequencing of colonic epithelial cells in a previous study [15] to select the genes co-expressed with ACE2, which are considered as potential therapeutic targets on COVID-19, and the gene names in the original files were standardized to match the human-related targets. The targets of the active ingredients of the drug and the targets of the disease were intersected, the intersectional targets were considered potential therapeutic targets of TJQW in COVID-19.

### Construction of a drug-active ingredient- potential target network

The components and the obtained active ingredients of TJQW and the target correspondence were imported into the Cytoscape (https://cytoscape.org/, version 3.7.0) [16] to construct the drug-active ingredient- potential target network.

### Network topology analysis of potential therapeutic targets

The Search Tool for the Retrieval of Interacting Genes/Proteins (STRING) (https://string-db.org/, version:11.0) [17], was used to construct a protein-protein interaction (PPI) network, the species were set to “*Homo sapiens*”; the lowest mutual action threshold was set to “medium confidence” (> 0.4), and other parameters were the default settings. And the PPI results were imported into cytoHubba (https://apps.cytoscape.org/apps/cytohubba, version 0.1) [18] for network topology analysis. An important hub gene can be selected through calculation and analysis of the network structure and the weighted reconnection between nodes. For each node in the interaction network, we selected “Degree” to calculate topological features, which is defined as the number of edges to node i. When applying the degree algorithm, the results were sorted by the degree value.

### Gene annotation analysis of potential therapeutic targets

The org. Hs.eg.db (https://www.bioconductor.org/, a package for the genome wide annotation of Human, primarily based on mapping using Entrez Gene identifiers, version 3.1.1) [19] and the clusterProfiler (https://www.bioconductor.org, a package implements methods to analyze and visualize functional profiles of gene and gene clusters, version 3.1.1) [20] on the R 3.5.2, were used to perform the gene ontology (GO) enrichment analysis [21] and the Kyoto Gene and Genome Encyclopedia (KEGG) enrichment analysis [22]. In our study, the R code is shown as below.

ClueGO (version 2.5.4) [23] and CluePedia (version 1.5.4) [24] plugins in Cytoscape were used to perform WikiPathways enrichment analysis. We chose the analysis mode of Functional analysis, load marker list is *Homo sapiens* (9606) and Symbol ID was input, then we selected “WikiPathways-503 terms/pathways with 6558 available unique genes-27.02.2019”. Regarding statistical options, we employed two-sided hypergeometric test and bonferroni step down for *p* value correction. With *P* < 0.05 were selected. The results of *p* value≤0.05 were used to graph the GO and KEGG pathways, illustrating the roles of targets and signaling pathways in COVID-19 treatment with TJQW.

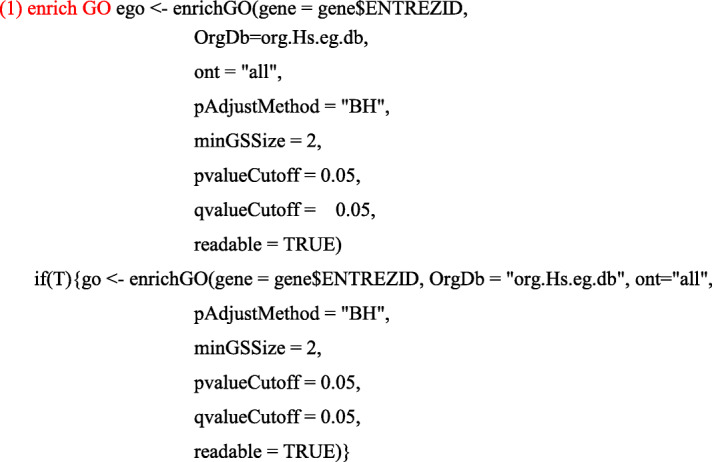

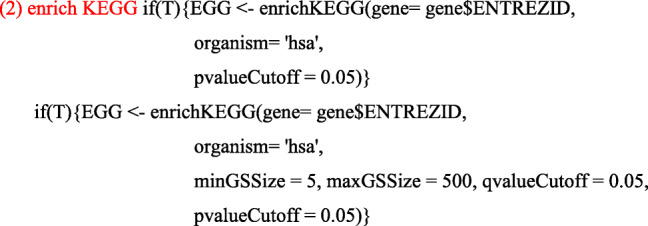





## Results

### Screening of the active ingredients in 16 components of TJQW

The TCMSP database was used to search for the active ingredients of TJQW, followed by using the parameters with ≥30% OB and ≥ 0.18 DL as the criteria to screen the 15 components (among all components, *Periostracum Cicadae* did not have any relevant retrieval results, which ingredients did not match the parameters with ≥30% OB and ≥ 0.18 DL) to obtain 238 active ingredients. Details of 15 components and its 238 active ingredients (without removing duplication) including herbal name, molecule ID, molecule name, OB information, DL information, and their CAS/InChIKey codes were shown in Supplementary material 1. After eliminating duplicates, a total of 194 active ingredients were obtained.

### Screening of active ingredients for COVID-19 related targets

**Fig. 2:**
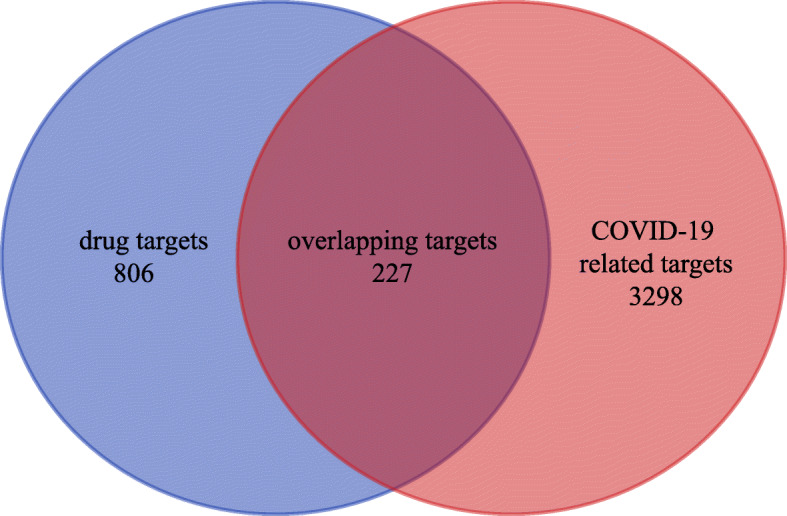
A total of 1033 and 3525 targets of TJQW and COVID-19, respectively, and they shared 227 targets

There are 38 out of 194 active ingredients that cannot be found in PubChem or cannot be predicted in Swiss TargetPrediction, so only 156 unique active ingredients and their target information are obtained, including a total of 1033 potential targets. According to the single-cell sequencing results of colonic epithelial cells, 5556 genes, which were co-expressed with ACE2, were identified. After normalizing the gene names in the original files, a total of 3525 human targets were matched. Subsequently, 1033 ingredient targets and 3525 COVID-19 targets were used to draw a Venn diagram to obtain 227 coincident targets, as shown in Fig. 1, i.e., the relevant targets of the TCM active ingredients acted on the disease. Moreover, the targets of 152 ingredients have an intersection with COVID-19, and 4 ingredients do not. Therefore, 148 potential active ingredients may be related to COVID-19, as shown in Fig. 2.

### The drug-active ingredient-target network analysis

**Fig. 3:**
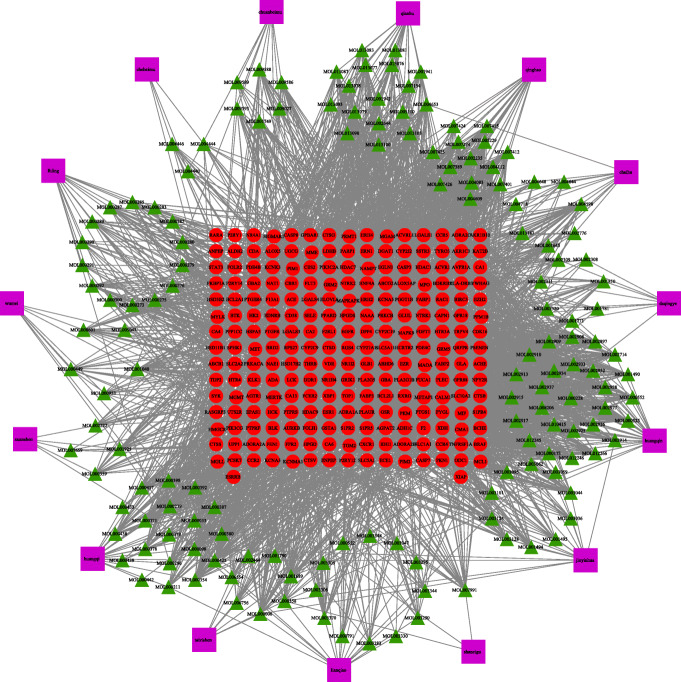
Drug-active ingredient-potential target network (purple color represents drugs, green color represents active ingredients, and red color represents potential targets, active ingredients are identified by molecular ID)

As shown in Fig. 3, Cytoscape was used to construct the drug-active ingredient-target network. On this network, different nodes represented drugs, potential active ingredients and effector targets of TJQW, and the network’s edges showed relationships between these three factors. Rectangles with purple color represent 15 components of TJQW. The surrounding green triangles represent their active ingredients. And the 227 red circles in the center represents the potential targets of TJQW on COVID-19. This fully indicated the characteristics of multi-components and multi-targets of TCM. When hub genes were found, a new simplified network was built to show relationships between key ingredients, key targets, and key pathways.

### Network topology analysis

**Fig. 4:**
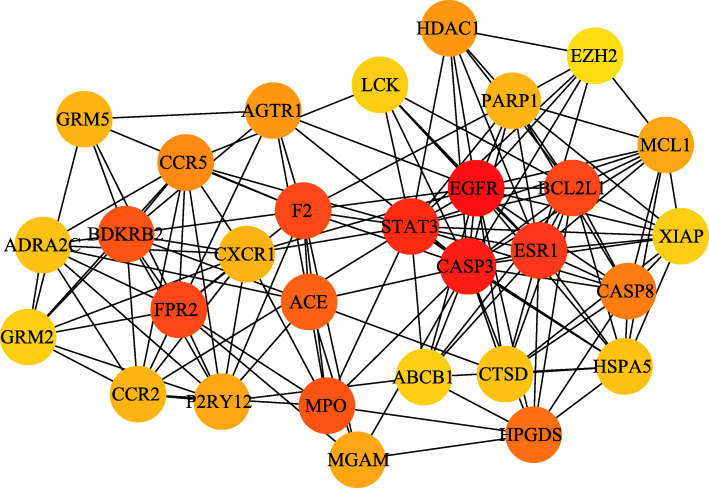
The PPI network of top 30 highest degree

The network topology analysis showed the network parameters with targets (Table 1 and Fig. 4), visually showing the network relationship of the top 30 targets with degree values. Figure 4 displays the connection status of these hub nodes in the network. The darker the color, the higher the value. There are 30 nodes and 155 edges in this network, average node degree is 28.8, the PPI enrichment *p*-value < 1.0e-16. The top ten targets are EGFR, CASP3, STAT3, ESR1, FPR2, F2, BCL2L1, BDKRB2, MPO, and ACE. Their source composition information was shown in Table 2.

**Table 1:** Top 30 in network ranked by Degree method

No.	Target	Degree	No.	Target	Degree
1	EGFR	63	16	MCL1	24
2	CASP3	54	17	MGAM	24
3	STAT3	46	18	P2RY12	24
4	ESR1	42	19	PARP1	23
5	BCL2L1	34	20	GRM5	23
6	F2	34	21	CXCR1	23
7	FPR2	34	22	CCR2	23
8	MPO	31	23	HSPA5	22
9	BDKRB2	31	24	CTSD	22
10	ACE	30	25	ADRA2C	22
11	HPGDS	28	26	XIAP	21
12	CASP8	27	27	LCK	21
13	CCR5	26	28	ABCB1	21
14	HDAC1	25	29	GRM2	21
15	AGTR1	25	30	EZH2	20

**Table 2:** Top ten targets and their source composition information

No	Target	Degree	Mol ID	Molecule Name	Probability^a^	OB (%)	DL	CAS/InChIKey	pinyin name	herbal name
1	EGFR	63	MOL000098	quercetin	1.000	46.43	0.28	117–39-5	wumei	Mume Fructus
									qinghao	*Artemisia annua* L.
									qianhu	Peucedani Radix
									lianqiao	Forsythiae Fructus
									jinyinhua	Lonicerae Japonicae Flos
									huangqi	*Hedysarum multijugum* Maxim.
									chaihu	Radix Bupleuri
			MOL000422	kaempferol	0.403	41.88	0.24	520–18-3	wumei	Mume Fructus
									qinghao	Artemisia Annua L.
									lianqiao	Forsythiae Fructus
									jinyinhua	Lonicerae Japonicae Flos
									huangqi	Hedysarum Multijugum Maxim.
									chaihu	Radix Bupleuri
			MOL000006	luteolin	0.271	36.16	0.25	491–70-3	taizishen	Pseudostellariae Radix
									qinghao	Artemisia Annua L.
									lianqiao	Forsythiae Fructus
									jinyinhua	Lonicerae Japonicae Flos
2	CASP3	54	MOL004653	(+)-Anomalin	0.119	46.06	0.66	PNTWXEIQXBRCP S-JLTIQLCOSA-N	chaihu	Radix Bupleuri
									qianhu	Peucedani Radix
			MOL013078	praeruptorin E	0.119	51.22	0.66	UFUVJROSOIXJGR -MKKZQTCBSA-N	qianhu	Peucedani Radix
			MOL013079	dl-praeruptorin a	0.116	46.46	0.53	73,069–25-7	qianhu	Peucedani Radix
3	STAT3	46	MOL003036	Stigmasterol Glucoside	0.181	43.83	0.76	19,716–26-8	jinyinhua	Lonicerae Japonicae Flos
			MOL002897	epiberberine	0.097	43.09	0.78	XXMJRBRPNZVNJ R-UHFFFAOYSA-N	huangqin	Scutellariae Radix
4	ESR1	42	MOL001040	(2R)-5,7-dihydroxy-2-(4-hydroxyphenyl)chroman-4-one	0.568	42.36	0.21	480–41-1	wumei	Mume Fructus
			MOL000006	luteolin	0.394	36.16	0.25	491–70-3	taizishen	Pseudostellariae Radix
									qinghao	Artemisia Annua L.
									lianqiao	Forsythiae Fructus
									jinyinhua	Lonicerae Japonicae Flos
			MOL000449	Stigmasterol	0.389	43.83	0.76	83–48-7	shancigu	Pseudobulbus Cremastrae Seu Pleiones
									jinyinhua	Lonicerae Japonicae Flos
									huangqin	Scutellariae Radix
									chaihu	Radix Bupleuri
									qinghao	Artemisia Annua L.
									wumei	Mume Fructus
5	FPR2	34	MOL013098	[(9R)-8,8-dimethyl-2-oxo-9,10-dihydropyrano[6,5-h]chromen-9-yl](Z)-2-methylbut-2-enoate	0.105	87.48	0.37	19,427–82-8	qianhu	Peucedani Radix
6	F2	34	MOL000371	3,9-di-Omethylnissolin	0.102	53.74	0.48	RFFNFQZKHNKOP O-BBRMVZONSAN	huangqi	Hedysarum Multijugum Maxim.
7	BCL2L1	34	MOL001749	ZINC03860434	0.116	43.59	0.35	BJQHLKABXJIVA M-WOJBJXKFSA-N	chuanbeimu	Fritiliariae Irrhosae Bulbus
			MOL001490	bis[(2S)-2-ethylhexyl] benzene-1,2-dicarboxylate	0.116	43.59	0.35	117–81-7	huangqin	Scutellariae Radix
			MOL013103	Umbelliprenin	0.113	46.57	0.44	532–16-1	qianhu	Peucedani Radix
8	BDKRB2	31	MOL013098	[(9R)-8,8-dimethyl-2-oxo-9,10-dihydropyrano[6,5-h]chromen-9-yl](Z)-2-methylbut-2-enoate	0.105	87.48	0.37	19,427–82-8	qianhu	Peucedani Radix
			MOL000371	3,9-di-Omethylnissolin	0.102	53.74	0.48	RFFNFQZKHNKOP O-BBRMVZONSAN	huangqi	Hedysarum Multijugum Maxim.
			MOL000398	isoflavanone	0.049	109.99	0.3	JNSVNRWHSLLCB G-LLVKDONJSA-N	huangqi	Hedysarum Multijugum Maxim.
9	MPO	31	MOL000098	quercetin	1.000	46.43	0.28	117–39-5	wumei	Mume Fructus
									qinghao	Artemisia Annua L.
									qianhu	Peucedani Radix
									lianqiao	Forsythiae Fructus
									jinyinhua	Lonicerae Japonicae Flos
									huangqi	Hedysarum Multijugum Maxim.
									chaihu	Radix Bupleuri
			MOL000422	kaempferol	0.394	41.88	0.24	520–18-3	wumei	Mume Fructus
									qinghao	Artemisia Annua L.
									lianqiao	Forsythiae Fructus
									jinyinhua	Lonicerae Japonicae Flos
									huangqi	Hedysarum Multijugum Maxim.
									chaihu	Radix Bupleuri
			MOL000006	luteolin	0.271	36.16	0.25	491–70-3	taizishen	Pseudostellariae Radix
									qinghao	Artemisia Annua L.
									lianqiao	Forsythiae Fructus
									jinyinhua	Lonicerae Japonicae Flos
10	ACE	30	MOL000273	(2R)-2-[(3S,5R,10S,13R,14R,16R,17R)-3,16-dihydroxy-4,4,10,13,14-pentamethyl-2,3,5,6,12,15,16,17-octahydro-1Hcyclopenta[a]phenanthren-17-yl]-6-methylhept-5-enoic acid	0.120	30.93	0.81	XSLKAKROJKMHI T-WIUKAADNSAN	fuling	Poria Cocos(Schw.) Wolf.
			MOL002308	Indicaxanthin	0.112	31.79	0.22	KYJMYFJJUHZAH X-QOBSUCFJSA-N	daqingye	Isatidis Folium
			MOL000280	(2R)-2-[(3S,5R,10S,13R,14R,16R,17R)-3,16-dihydroxy-4,4,10,13,14-pentamethyl-2,3,5,6,12,15,16,17-octahydro-1Hcyclopenta[a]phenanthren-17-yl]-5-isopropyl-hex-5-enoic acid	0.111	31.07	0.82	6754-16-1	fuling	Poria Cocos(Schw.) Wolf.

^a^Probability value from Swiss TargetPrediction for the query molecule to have this protein as target

### GO enrichment analysis

**Fig. 5:**
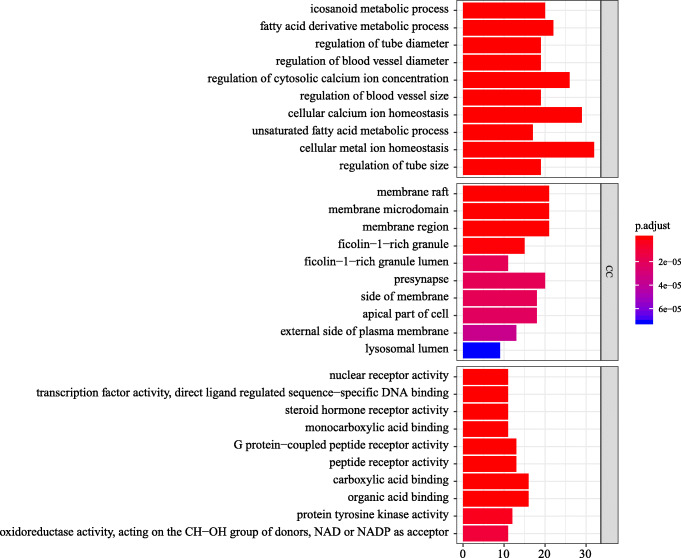
The GO enrichment analysis (*P* < 0.05)

GO enrichment analysis results included GO-molecular function (MF), GO-biological process (BP), and GO-cell component (CC), followed by the selection of the top 10 enrichment results. In Fig. 5, the y-axis represents the enriched categories and the x-axis represents the number of enrichment. The top 10 results of GO-BP included blood vessel diameter regulation, blood vessel inner diameter regulation, cytosolic calcium-ion concentration regulation, cellular calcium-ion homeostasis, intracellular metal-ion hemostasis, and unsaturated fatty-acid metabolism process. The top 10 enrichment results of GO-CC included membrane rafts, membrane micro-regions, membrane regions, presynapses, membrane sides, apical part of the cell, plasma membrane outer surface, and lysosome cavity. The top 10enrichment results of GO-MF included nuclear receptor activity, steroid hormone receptor activity, monocarboxylic acid-binding, G protein-coupled receptor activity, peptide receptor activity, organic acid binding, protein tyrosine kinase activity, and oxidoreductase activity.

### Signaling pathways enrichment analysis

**Fig. 6:**
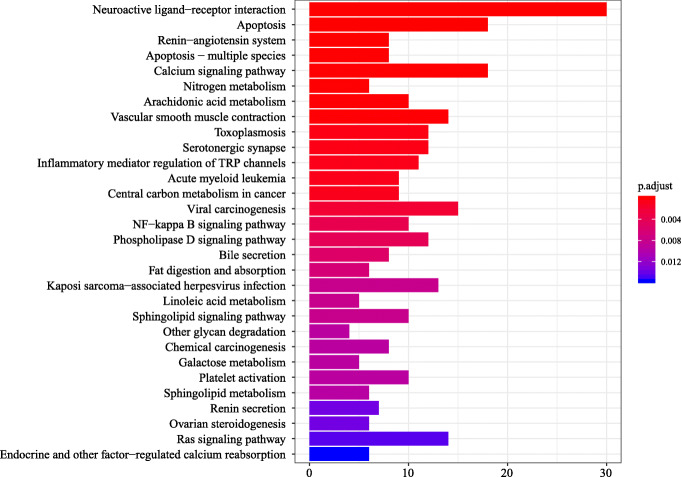
The KEGG pathways enrichment analysis (*P* < 0.05)

KEGG pathways enrichment analysis was shown in Fig. 6, the y-axis represents the enrichment pathway name and the x-axis represents the numbers of enrichment. The results suggested that the KEGG pathways of TJQW targets against COVID-19 were mainly involved in neuroactive ligand-receptor interaction, apoptosis, renin-angiotensin system, calcium signaling pathway, arachidonic acid metabolism, vascular smooth muscle contraction, toxoplasmosis, Inflammatory mediator regulation of transient receptor potential (TRP) channels, acute myeloid leukemia, central carbon metabolism in cancer, platelet activation, and nuclear factor kappa-B (NF-κB) signaling pathway.

**Fig. 7:**
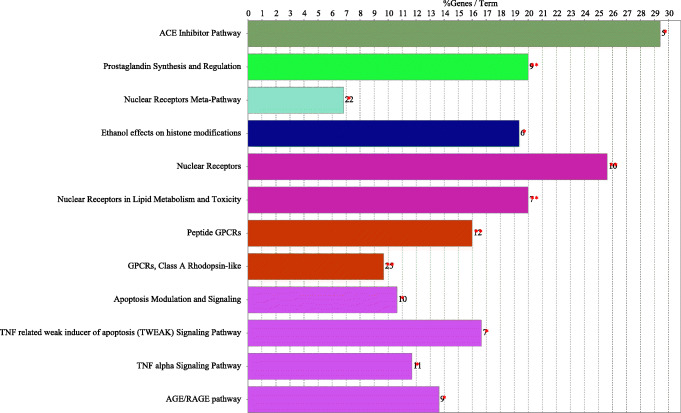
WikiPathways enrichment analysis (*P* < 0.05)

**Fig. 8:**
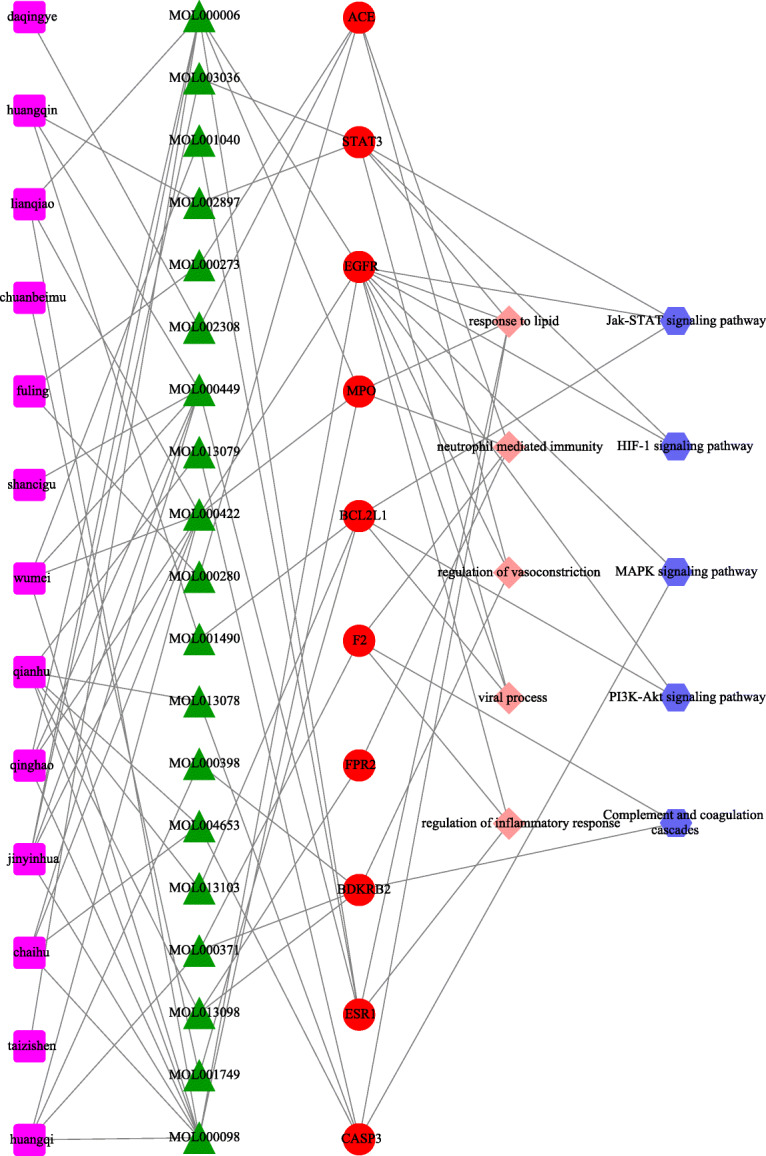
Key network of “drug-component-target-biological process- signaling pathway” (*P* < 0.05)

WikiPathways enrichment analysis was shown in Fig. 7, the y-axis represents the enrichment pathway name and the x-axis represents the numbers of enrichment. The enriched WikiPathways (*P* < 0.05) were involved in ACE Inhibitor pathway, prostaglandin synthesis and regulation, nuclear receptors meta-pathway, ethanol effects on histone modifications, nuclear receptors in lipid metabolism and toxicity, apoptosis modulation and signaling, TNF related weak inducer of apoptosis (TWEAK) signaling pathway, TNF alpha signaling pathway, and AGE/RAGE pathway. Furthermore, based on the above comprehensive analysis, the key network of “drug-component-target-biological process- signaling pathway” was presented (Fig. 8).

## Discussion

Clinical research shows that integrative therapy by combining TCM and western medicine for COVID-19 is significantly better at improving the symptoms and shortening the average length of hospital stay in COVID-19 patients than treatments using western medicine [25]. Early intervention using TCM reduces the transition from moderate to severe and critical COVID-19. The treatment strategy of TCM focuses on eliminating fever and expelling pathogenic factors, especially in relieving endogenous stagnated heat in the body to thus eliminate the pathogens [26]. Considering the characteristics of humid and hot climate of Lingnan area, supplementation with heat and damp-clearing agents is needed. In addition, TCM classic, *Huangdi* 81 *Nanjing* claims that “individuals with lung injuries are benefited from *qi* tonification.” Tonifying *qi* and nourishing *yin* in the early stage of COVID-19 to balance and maintain the vital energy is necessary.

Thus, the TJQW were formulated, with an emphasis on using heat-clearing and detoxifying herbs, such as *Forsythiae Fructus*, edible tulip, *Lonicera japonica*, *Radix Scutellariae baicalensis*, and *Folium Isatidis*, combined with *Fritillaria cirrhosa* and *Fritillaria thunbergii* for heat-clearing and phlegm removal, *Poria cocos* for spleen invigoration and dampness elimination, radix *Scrophulariae* for yin nourishment, and *Fructus Mume* as an astringent herb for stabilizing qi in the lungs. Combination of the two herbs was used to calm the body parts that were not affected by the pathogenic factors. *Radix Pseudostellariae* and *Astragalus propinquus* were used to balance and maintain the vital energy and eliminate the pathogenic factors. Supplementation of herbs against cold-nature and to maintain the vital energy also relieved the *qi* and *yin* deficiency in the later stage of the disease [3].

Prescription of TJQW has been incorporated into the TCM treatment program for COVID-19 in Guangdong Province [18]. At this time, no high-quality evidence supports the clinical efficacy of TJQW treatment against COVID-19. However, we can perform bioinformatics analysis as a theoretical evaluation of potential anti-COVID-19 molecular mechanisms of TJQW to support future clinical trials. This study used network pharmacology to explore the potential mechanism of COVID-19 treatment with TJQW, and the findings are summarized below.

### Active ingredients

In the analysis of TJQW-active ingredient-potential target network, the active ingredients, including umbelliprenin, quercetin, kaempferol, luteolin, praeruptorin E, stigmasterol, and oroxylin A, have potential anti-inflammatory and antiviral effects.

Biological activities including anti-inflammatory, antioxidant, and antileishmanial activities have been reported from umbelliprenin (UMB) [27], UMB induced T(H)2 IL-4 and suppressed T(H)1 IFNγ secretion, and significantly suppressed LPS-induced production of NO and PGE (2) apparently and also led to reductions in inducible iNOS and COX-expression [28], showing anti-inflammatory and immunomodulatory properties in vitro [29]. Numerous studies on quercetin and quercetin glycosides have been proved that these compounds acts as potent drug candidate towards inhibition of influenza virus infection through inferring the viral cellular immune system, inhibition of viral cellular targets, interfering the viral replication and inhibiting the viral growth phases [30]. Kaempferol suppressed the expression of proinflammatory cytokine interleukin-6 and chemokines interleukin-8, monocyte chemoattractant protein-1, and regulated on activation, normal T-cell expressed and secreted [31]. Luteolin inhibits LPS-induced inflammatory responses through modulation of NF-κB/AP-1/PI3K-Akt signaling cascades [32]. Luteolin decreased the expression of core genes at protein and mRNA levels (MMP9, MAPK1, HSP90AA1, EGFR, SRC and HRAS), and PI3K-AKT signaling pathway, Ras signaling pathway might be the critical pathways of luteolin against inflammation [33]. In silico, in vitro, in vivo, and clinical studies strongly suggest that the major pharmacological mechanism of luteolin is its anti-inflammatory activity, which derives from its regulation of transcription factors such as STAT3 and NF-κB [34]. Praeruptorin E suppressed lipopolysaccharide (LPS) induced Nuclear Factor-kappa B (NF-κB) pathway activation in the lung by decreasing the cytoplasmic loss of Inhibitor κB-α (IκB-α) protein and inhibiting the translocation of p65 from the cytoplasm to nucleus, which might be useful in the therapy of lung injury [35]. Treatment of stigmasterol significantly suppresses the expression of proinflammatory mediators (TNF-α, IL-6, IL-1β, iNOS and COX-2) and increases the expression of anti-inflammatory cytokine (IL-10) [36]. Based on an in silico approach, an extensive molecular docking investigation of the phytocompounds at the active binding pockets of the viral proteins revealed the promising inhibitory potential on COVID-19 of stigmasterol [37]. Vascular dysfunction plays a critical role in the pathogenesis of sepsis in COVID-19. Oroxylin A regulates the vascular tone by inhibiting vascular hyporeactivity caused by NO overproduction and reverses the endothelial barrier dysfunction and inflammation by inhibiting the IRAK-4-mediated IKKα/β phosphorylation, suggesting that Oroxylin A administration as a potentially useful therapeutic approach for clinical interventions in septic shock [38].

### Potential therapeutic mechanism

In terms of potential therapeutic targets, based on this study, all potential biotargets of TJQW action against COVID-19 were methodically obtained, and the potential core pharmacological targets were identified as EGFR, CASP3, STAT3, ESR1, FPR2, F2, BCL2L1, BDKRB2, MPO, and ACE, majority of them have been confirmed to be involved in pulmonary inflammation. EGFR-mediated signaling pathways are important for cell proliferation, differentiation, and survival in many tissues and cell types. Suppression of the EGFR pathway via the reduction of eIF2α phosphorylation increases susceptibility to cellular oxidative stress [39]. The parameters including homocysteine, urea, creatinine and serum cystatin C were significantly higher in imaging progression patients, while EGFR decreased [40]. This suggested that low levels of EGFR may be a protective factor for COVID-19. Caspases are a family of enzymes that play a key role in apoptosis. They are mediators and performers of apoptosis. When CASP8 interacts with CASP3, CASP3 is activated and it further induces apoptosis [41]. *Radix Scutellariae baicalensis* inhibits the apoptosis induced by influenza virus infection through regulating the expression of apoptosis-related genes in the CASP8-mediated exogenous pathway and the pathways of endoplasmic reticulum [42]. ACE2, a SARS-CoV-2 receptor, is upregulated by interleukin-6 via STAT3 signaling in synovial tissues [43]. In the later phase of COVID-19, the potential dysregulation of the AngII-AT1R pathway downstream of ACE2 could lead to cytokine release syndrome, and the IL-6-STAT3 axis may be the required targeting of cytokine pathways in the treatment in COVID-19 patients [44]. STAT3 and BCL2L1 are considered to have protein interactions with SARS-CoV [45]. A critical imbalance in RAS represented by decreased expression of ACE in combination with increases in ACE2 and both bradykinin receptors (BDKRB1, BDKRB2) [46].

Estrogen receptor decreases NF-kappa-B DNA-binding activity and inhibits NF-kappa-B-mediated transcription from the IL6 promoter [47]. The human FPR2, which is powerful neutrophils chemotactic factor, plays a crucial role in host defense and inflammation, and has been considered as a drug target for chronic inflammatory diseases [48]. Accumulating evidence demonstrates that FPR2 are critical mediators of myeloid cell trafficking in the sequential chemotaxis signal relays in microbial infection, inflammation, and immune responses [49]. As receptor, FPR2 displays potent mucosal protection and promotes catabasis after acute lung injury (ALI) [50], providing a novel therapeutic target to develop an effective treatment against ALI progression. F2 has functions in blood homeostasis, inflammation and wound healing [51]. Antiapoptotic proteins Bcl-2 and Bcl-X(L) may attenuate inflammation impairing NLRP1-inflammasome activation, reducing caspase-1 activation and interleukin-1beta (IL-1beta) production [52]. The increasing expression of Bcl-2 and Bcl-xL inhibits autophagy and bacterial killing in human macrophages, suggesting that targeting the Bcl-2/Bcl-xL-Beclin 1 interaction may improving resistance to infection in patients with advanced lung disease [53]. MPO is a part of the host defense system of polymorphonuclear leukocytes, and it is responsible for microbicidal activity against a wide range of organisms [54, 55].

The bioinformatics findings of this study suggested that the specific inactivation of signaling pathways of inflammatory stress and targeted modulation of intrapulmonary EGFR, CASP3, STAT3, ESR1, FPR2, F2, BCL2L1, BDKRB2, MPO, and ACE expressions, maybe potential pathways of TJQW action against COVID-19. These are the potential therapeutic mechanisms of active ingredients in TJQW and targets in the treatment of COVID-19. This study provided new predictions on the molecular-based therapeutic mechanism of TJQW in COVID-19 treatment and it offered new ideas for further study.

According to the results of GO enrichment analysis, the potential therapeutic targets of TJQW were mainly distributed in the regulation of inflammatory response, viral process, neutrophil mediated immunity, response to lipid, regulation of vasoconstriction, and were also involved in promotion/suppression of inflammation, cell division, abnormal cell proliferation, oxidation/reduction reactions, ion-channel regulation, and immune regulation. From the results of KEGG enrichment analysis, the pathways mainly involved in the treatment process, PI3K-Akt signaling pathway, MAPK signaling pathway, Jak-STAT signaling pathway, Complement and coagulation cascades, and HIF-1 signaling pathway, were important inflammatory or metabolism pathways.

This study implicated MAPK signaling as part of the potential mechanisms underlying TJQW antiviral function. MAPK cascades are crucial signaling pathways in the regulation of host immune response to infection [56]. MAPK phosphatase 1 regulates pro- and anti-inflammatory cytokines dynamically in innate immune responses [57]. Studies suggest that quercetin possesses antioxidant, anti-inflammatory, and antiplatelet properties, Quercetin-mediated antiplatelet activity involves PI3K/Akt inactivation, suppresses MAPK phosphorylations. Quercetin may have the potential to treat diseases involving aberrant platelet activation and inflammation [58]. Patients with severe COVID-19 might experience cytokine release syndrome, and several of the cytokines involved in COVID-19 employ a distinct intracellular signaling pathway mediated by Janus kinases (JAKs), therefore, JAK inhibition presents an attractive therapeutic strategy for CRS [59]. What’s more, it has been reported that complement and HIF-1 are potential pathways of damage and targets for the cure of COVID-19-driven endothelial damage [60].

Besides, the arachidonic acid metabolic pathway is mainly used for the synthesis of inflammatory mediators, which mediate the production of various inflammatory cytokines, such as monocyte chemotactic-protein-1, TNF, IL, and interferon, which are related to the development, progression, and regression of inflammation [61]. In addition to regulating blood pressure and water and sodium balance in the renin angiotensin system (RAS), a study has shown that the system is also involved in regulating various physiological activities, such as inflammation, immunity, apoptosis, and tissue repair in the body. In addition, it is also involved in the development and progression of lung diseases, such as pulmonary arterial hypertension, pulmonary fibrosis, and pulmonary thromboembolism, especially acute lung injury [62].

These findings suggested that TJQW may exert antioxidative, antiviral, and anti-inflammatory effects, along with immune system activation effects through the aforementioned targets, thereby playing an early protective role in the lungs. While in the TCM treatment program for COVID-19 in Guangdong Province, TJQW was mainly used for the treatment of COVID-19 in the early stage.

In summary, this study used network pharmacology techniques and approach to retrieve the active ingredients and potential targets of TCM in TJQW, by screening the common targets for TCM active ingredients and diseases and performing GO and KEGG enrichment analyses of the targets. As shown in the key network of “drug-component-target-biological process- signaling pathway”, we speculated that the active ingredients in the TJQW treatment of COVID-19 may be umbelliprenin, quercetin, luteolin, praeruptorin E, stigmasterol. The potential effector targets were EGFR, CASP3, STAT3, ESR1, FPR2, F2, BCL2L1, BDKRB2, MPO, and ACE, through regulating signaling pathways, such as PI3K-Akt signaling pathway, MAPK signaling pathway, Jak-STAT signaling pathway, to suppress the inflammatory response, regulate the immune function, and reduce lung injury, thereby achieving the purpose of COVID-19 treatment.

From the perspective of research limitations. Since the gene co-expressed with ACE2 was selected as a potential therapeutic target for COVID-19 in this study, it is not a specific therapeutic target and needs further study.

## Conclusion

Our study uncovered the pharmacological mechanism underlying TJQW treatment for COVID-19. These results should benefit efforts for people around the world to gain more knowledge about Chinese medicine TJQW in the treatment of this vicious epidemic COVID-19, and help to address this pressing problem currently facing the world.

## Supplementary information

**Additional file 1.**

## Data Availability

All data are available in the manuscript and the they are showed in tables, figures.
