# Supplementary material for: Therapeutic mechanism of Toujie Quwen granules in COVID-19 based on network pharmacology
Source: BioData Min. 2020 Sep 24;13:15. doi: 10.1186/s13040-020-00225-8 (PMC7512049; doi:10.1186/s13040-020-00225-8)
Supplement: Supplementary file 1 — Additional file 1. [file 13040_2020_225_MOESM1_ESM.docx]

**Supplementary material 1**

**Table 1. Details of 15 components and its 238 active ingredients**

There are 38 active ingredients that cannot be found in PubChem or cannot be predicted in Swiss were marked “**^*^**”.

| No. | pinyin name | herbal name | Mol ID | Molecule Name | OB (%) | DL | CAS/InChIKey |
| --- | --- | --- | --- | --- | --- | --- | --- |
| 1 | lianqiao | Forsythiae Fructus | MOL000006 | luteolin | 36.16 | 0.25 | 491-70-3 |
| 2 | lianqiao | Forsythiae Fructus | MOL000098 | quercetin | 46.43 | 0.28 | 117-39-5 |
| 3 | lianqiao | Forsythiae Fructus | MOL000173 | wogonin | 30.68 | 0.23 | 632-85-9 |
| 4 | lianqiao | Forsythiae Fructus | MOL000211 | Mairin | 55.38 | 0.78 | 472-15-1 |
| 5 | lianqiao | Forsythiae Fructus | MOL000358 | beta-sitosterol | 36.91 | 0.75 | 83-46-5 |
| 6 | lianqiao | Forsythiae Fructus | MOL000422 | kaempferol | 41.88 | 0.24 | 520-18-3 |
| 7 | lianqiao | Forsythiae Fructus | MOL000522 | arctiin | 34.45 | 0.84 | 20362-31-6 |
| 8 | lianqiao | Forsythiae Fructus | MOL000791 | bicuculline | 69.67 | 0.88 | 485-49-4 |
| 9^*^ | lianqiao | Forsythiae Fructus | MOL003281 | 20(S)-dammar-24-ene-3β,20-diol-3-acetate | 40.23 | 0.82 | QSUGODIDPUGIFV-SJPISLFHSA-N |
| 10 | lianqiao | Forsythiae Fructus | MOL003283 | (2R,3R,4S)-4-(4-hydroxy-3-methoxy-phenyl)-7-methoxy-2,3-dimethylol-tetralin-6-ol | 66.51 | 0.39 | 548-29-8 |
| 11 | lianqiao | Forsythiae Fructus | MOL003290 | (3R,4R)-3,4-bis[(3,4-dimethoxyphenyl)methyl]oxolan-2-one | 52.3 | 0.48 | 17283-81-7 |
| 12 | lianqiao | Forsythiae Fructus | MOL003295 | (+)-pinoresinol monomethyl ether | 53.08 | 0.57 | CPJKKWDCUOOTEW-LYDRAKHJSA-N |
| 13 | lianqiao | Forsythiae Fructus | MOL003305 | PHILLYRIN | 36.4 | 0.86 | KFFCKOBAHMGTMW-AJBQBZEWSA-N |
| 14 | lianqiao | Forsythiae Fructus | MOL003306 | ACon1_001697 | 85.12 | 0.57 | CPJKKWDCUOOTEW-VUEDXXQZSA-N |
| 15^*^ | lianqiao | Forsythiae Fructus | MOL003308 | (+)-pinoresinol monomethyl ether-4-D-beta-glucoside_qt | 61.2 | 0.57 | DJHDOMQWAUJNKX-SORVUCRQSA-N |
| 16^*^ | lianqiao | Forsythiae Fructus | MOL003315 | 3beta-Acetyl-20,25-epoxydammarane-24alpha-ol | 33.07 | 0.79 | TVDGNOQUKHVJSM-KUPITCAKSA-N |
| 17^*^ | lianqiao | Forsythiae Fructus | MOL003322 | FORSYTHINOL | 81.25 | 0.57 | IBDUMNIUXZHNCQ-LVNJIZSUSA-N |
| 18 | lianqiao | Forsythiae Fructus | MOL003330 | (-)-Phillygenin | 95.04 | 0.57 | 487-39-8 |
| 19 | lianqiao | Forsythiae Fructus | MOL003344 | β-amyrin acetate | 42.06 | 0.74 | UMRPOGLIBDXFNK-OCPHCQIBSA-N |
| 20 | lianqiao | Forsythiae Fructus | MOL003347 | hyperforin | 44.03 | 0.6 | 11079-53-1 |
| 21 | lianqiao | Forsythiae Fructus | MOL003348 | adhyperforin | 44.03 | 0.61 | 143183-63-5 |
| 22^*^ | lianqiao | Forsythiae Fructus | MOL003365 | Lactucasterol | 40.99 | 0.85 | CYEIJRKSMYAHGE-XRTCGMRYSA-N |
| 23 | lianqiao | Forsythiae Fructus | MOL003370 | Onjixanthone I | 79.16 | 0.3 | PLTRADDVONOEAX-UHFFFAOYSA-N |
| 24 | shancigu | Pseudobulbus Cremastrae Seu Pleiones | MOL000358 | beta-sitosterol | 36.91 | 0.75 | 83-46-5 |
| 25 | shancigu | Pseudobulbus Cremastrae Seu Pleiones | MOL000449 | Stigmasterol | 43.83 | 0.76 | 83-48-7 |
| 26 | shancigu | Pseudobulbus Cremastrae Seu Pleiones | MOL007991 | 2-methoxy-9,10-dihydrophenanthrene-4,5-diol | 44.97 | 0.18 | 70205-50-4 |
| 27 | jinyinhua | Lonicerae Japonicae Flos | MOL000006 | luteolin | 36.16 | 0.25 | 491-70-3 |
| 28 | jinyinhua | Lonicerae Japonicae Flos | MOL000098 | quercetin | 46.43 | 0.28 | 117-39-5 |
| 29 | jinyinhua | Lonicerae Japonicae Flos | MOL000358 | beta-sitosterol | 36.91 | 0.75 | 83-46-5 |
| 30 | jinyinhua | Lonicerae Japonicae Flos | MOL000422 | kaempferol | 41.88 | 0.24 | 520-18-3 |
| 31 | jinyinhua | Lonicerae Japonicae Flos | MOL000449 | Stigmasterol | 43.83 | 0.76 | 83-48-7 |
| 32 | jinyinhua | Lonicerae Japonicae Flos | MOL001494 | Mandenol | 42 | 0.19 | 544-35-4 |
| 33 | jinyinhua | Lonicerae Japonicae Flos | MOL001495 | Ethyl linolenate | 46.1 | 0.2 | JYYFMIOPGOFNPK-XSHSMGBESA-N |
| 34^*^ | jinyinhua | Lonicerae Japonicae Flos | MOL002707 | phytofluene | 43.18 | 0.5 | OVSVTCFNLSGAMM-BBVOSDEVSA-N |
| 35 | jinyinhua | Lonicerae Japonicae Flos | MOL002773 | beta-carotene | 37.18 | 0.58 | 7235-40-7 |
| 36 | jinyinhua | Lonicerae Japonicae Flos | MOL002914 | Eriodyctiol (flavanone) | 41.35 | 0.24 | 4049-38-1 |
| 37^*^ | jinyinhua | Lonicerae Japonicae Flos | MOL003006 | (-)-(3R,8S,9R,9aS,10aS)-9-ethenyl-8-(beta-D-glucopyranosyloxy)-2,3,9,9a,10,10a-hexahydro-5-oxo-5H,8H-pyrano[4,3-d]oxazolo[3,2-a]pyridine-3-carboxylic acid_qt | 87.47 | 0.23 | CJISYUMYHKJZBF-HZOXHFDSSA-N |
| 38^*^ | jinyinhua | Lonicerae Japonicae Flos | MOL003014 | secologanic dibutylacetal_qt | 53.65 | 0.29 | MQMIOCHUWUGSPS-LLGFUMIMSA-N |
| 39 | jinyinhua | Lonicerae Japonicae Flos | MOL003036 | ZINC03978781 | 43.83 | 0.76 | 19716-26-8 |
| 40 | jinyinhua | Lonicerae Japonicae Flos | MOL003044 | Chryseriol | 35.85 | 0.27 | 491-71-4 |
| 41 | jinyinhua | Lonicerae Japonicae Flos | MOL003059 | kryptoxanthin | 47.25 | 0.57 | 472-70-8 |
| 42 | jinyinhua | Lonicerae Japonicae Flos | MOL003062 | 4,5'-Retro-.beta.,.beta.-Carotene-3,3'-dione, 4',5'-didehydro- | 31.22 | 0.55 | 116-30-3 |
| 43 | jinyinhua | Lonicerae Japonicae Flos | MOL003095 | 5-hydroxy-7-methoxy-2-(3,4,5-trimethoxyphenyl)chromone | 51.96 | 0.41 | 18103-41-8 |
| 44 | jinyinhua | Lonicerae Japonicae Flos | MOL003101 | 7-epi-Vogeloside | 46.13 | 0.58 | SJTKENDJEOSSGF-ZPQFOYGRSA-N |
| 45^*^ | jinyinhua | Lonicerae Japonicae Flos | MOL003108 | Caeruloside C | 55.64 | 0.73 | UBFWADWFXZXMGP-VQVVDCOFSA-N |
| 46^*^ | jinyinhua | Lonicerae Japonicae Flos | MOL003111 | Centauroside_qt | 55.79 | 0.5 | LTDRLWUNCZGQEM-NBWBJBPESA-N |
| 47^*^ | jinyinhua | Lonicerae Japonicae Flos | MOL003117 | Ioniceracetalides B_qt | 61.19 | 0.19 | HOJAQTBWHRNJPB-XKJBLICVSA-N |
| 48 | jinyinhua | Lonicerae Japonicae Flos | MOL003124 | XYLOSTOSIDINE | 43.17 | 0.64 | RMTHFMSBPWQULL-QQHNMQKUSA-N |
| 49 | jinyinhua | Lonicerae Japonicae Flos | MOL003128 | dinethylsecologanoside | 48.46 | 0.48 | 25488-59-9 |
| 50^*^ | huangqin | Scutellariae Radix | MOL000073 | ent-Epicatechin | 48.96 | 0.24 | 35323-91-2 |
| 51 | huangqin | Scutellariae Radix | MOL000173 | wogonin | 30.68 | 0.23 | 632-85-9 |
| 52 | huangqin | Scutellariae Radix | MOL000228 | (2R)-7-hydroxy-5-methoxy-2-phenylchroman-4-one | 55.23 | 0.2 | QQQCWVDPMPFUGF-CYBMUJFWSA-N |
| 53 | huangqin | Scutellariae Radix | MOL000358 | beta-sitosterol | 36.91 | 0.75 | 83-46-5 |
| 54 | huangqin | Scutellariae Radix | MOL000359 | sitosterol | 36.91 | 0.75 | KZJWDPNRJALLNS-ZFVHJZABSA-N |
| 55 | huangqin | Scutellariae Radix | MOL000449 | Stigmasterol | 43.83 | 0.76 | 83-48-7 |
| 56 | huangqin | Scutellariae Radix | MOL000525 | Norwogonin | 39.4 | 0.21 | 4443-9—8 |
| 57 | huangqin | Scutellariae Radix | MOL000552 | 5,2'-Dihydroxy-6,7,8-trimethoxyflavone | 31.71 | 0.35 | 86926-52-5 |
| 58^*^ | huangqin | Scutellariae Radix | MOL001458 | coptisine | 30.67 | 0.86 | RMOPHAINWJSBTC-UHFFFAOYSA-N |
| 59 | huangqin | Scutellariae Radix | MOL001490 | bis[(2S)-2-ethylhexyl] benzene-1,2-dicarboxylate | 43.59 | 0.35 | 117-81-7 |
| 60 | huangqin | Scutellariae Radix | MOL001506 | Supraene | 33.55 | 0.42 | 111-02-4 |
| 61 | huangqin | Scutellariae Radix | MOL001689 | acacetin | 34.97 | 0.24 | 480-44-4 |
| 62 | huangqin | Scutellariae Radix | MOL002714 | baicalein | 33.52 | 0.21 | 491-67-8 |
| 63 | huangqin | Scutellariae Radix | MOL002879 | Diop | 43.59 | 0.39 | 27554-26-3 |
| 64 | huangqin | Scutellariae Radix | MOL002897 | epiberberine | 43.09 | 0.78 | XXMJRBRPNZVNJR-UHFFFAOYSA-N |
| 65 | huangqin | Scutellariae Radix | MOL002908 | 5,8,2'-Trihydroxy-7-methoxyflavone | 37.01 | 0.27 | 77056-20-3 |
| 66 | huangqin | Scutellariae Radix | MOL002909 | 5,7,2,5-tetrahydroxy-8,6-dimethoxyflavone | 33.82 | 0.45 | XGJUUNRZFPFMOK-UHFFFAOYSA-N |
| 67 | huangqin | Scutellariae Radix | MOL002910 | Carthamidin | 41.15 | 0.24 | 479-54-9 |
| 68^*^ | huangqin | Scutellariae Radix | MOL002911 | 2,6,2',4'-tetrahydroxy-6'-methoxychaleone | 69.04 | 0.22 | XFUBPGKNMBCAHX-WAYWQWQTSA-N |
| 69 | huangqin | Scutellariae Radix | MOL002913 | Dihydrobaicalin_qt | 40.04 | 0.21 | GPDJGLOROGNHJD-NSHDSACASA-N |
| 70 | huangqin | Scutellariae Radix | MOL002914 | Eriodyctiol (flavanone) | 41.35 | 0.24 | 4049-38-1 |
| 71 | huangqin | Scutellariae Radix | MOL002915 | Salvigenin | 49.07 | 0.33 | 19103-54-9 |
| 72 | huangqin | Scutellariae Radix | MOL002917 | 5,2',6'-Trihydroxy-7,8-dimethoxyflavone | 45.05 | 0.33 | 92519-93-2 |
| 73 | huangqin | Scutellariae Radix | MOL002925 | 5,7,2',6'-Tetrahydroxyflavone | 37.01 | 0.24 | 82475-00-1 |
| 74 | huangqin | Scutellariae Radix | MOL002926 | dihydrooroxylin A | 38.72 | 0.23 | 18956-18-8 |
| 75 | huangqin | Scutellariae Radix | MOL002927 | Skullcapflavone II | 69.51 | 0.44 | 55084-08-7 |
| 76 | huangqin | Scutellariae Radix | MOL002928 | oroxylin a | 41.37 | 0.23 | 480-11-5 |
| 77 | huangqin | Scutellariae Radix | MOL002932 | Panicolin | 76.26 | 0.29 | 41060-16-6 |
| 78 | huangqin | Scutellariae Radix | MOL002933 | 5,7,4'-Trihydroxy-8-methoxyflavone | 36.56 | 0.27 | 57096-02-3 |
| 79 | huangqin | Scutellariae Radix | MOL002934 | NEOBAICALEIN | 104.34 | 0.44 | 55084-08-7 |
| 80 | huangqin | Scutellariae Radix | MOL002937 | DIHYDROOROXYLIN | 66.06 | 0.23 | 18956-18-8 |
| 81 | huangqin | Scutellariae Radix | MOL008206 | Moslosooflavone | 44.09 | 0.25 | 3570-62-5 |
| 82 | huangqin | Scutellariae Radix | MOL010415 | 11,13-Eicosadienoic acid, methyl ester | 39.28 | 0.23 | QKVKDCGPQOJFNM-BNFZFUHLSA-N |
| 83 | huangqin | Scutellariae Radix | MOL012245 | 5,7,4'-trihydroxy-6-methoxyflavanone | 36.63 | 0.27 | 94942-49-1 |
| 84 | huangqin | Scutellariae Radix | MOL012246 | 5,7,4'-trihydroxy-8-methoxyflavanone | 74.24 | 0.26 | 57096-02-3 |
| 85 | huangqin | Scutellariae Radix | MOL012266 | rivularin | 37.94 | 0.37 | 70028-59-0 |
| 86 | daqingye | Isatidis Folium | MOL000358 | beta-sitosterol | 36.91 | 0.75 | 83-46-5 |
| 87 | daqingye | Isatidis Folium | MOL001771 | poriferast-5-en-3beta-ol | 36.91 | 0.75 | KZJWDPNRJALLNS-FBZNIEFRSA-N |
| 88 | daqingye | Isatidis Folium | MOL001781 | Indigo | 38.2 | 0.26 | COHYTHOBJLSHDF-BUHFOSPRSA-N |
| 89 | daqingye | Isatidis Folium | MOL001810 | 6-(3-oxoindolin-2-ylidene)indolo[2,1-b]quinazolin-12-one | 45.28 | 0.89 | 97457-31-3 |
| 90 | daqingye | Isatidis Folium | MOL002308 | Indicaxanthin | 31.79 | 0.22 | KYJMYFJJUHZAHX-QOBSUCFJSA-N |
| 91 | daqingye | Isatidis Folium | MOL002309 | indirubin | 48.59 | 0.26 | 479-41-4 |
| 92 | daqingye | Isatidis Folium | MOL002311 | Glycyrol | 90.78 | 0.67 | 23013-84-5 |
| 93^*^ | daqingye | Isatidis Folium | MOL002318 | C05837 | 66.02 | 0.48 | 4356-52-9 |
| 94 | daqingye | Isatidis Folium | MOL002320 | γ-sitosterol | 36.91 | 0.75 | 83-47-6 |
| 95 | daqingye | Isatidis Folium | MOL002322 | isovitexin | 31.29 | 0.72 | MYXNWGACZJSMBT-VJXVFPJBSA-N |
| 96 | chaihu | Radix Bupleuri | MOL000098 | quercetin | 46.43 | 0.28 | 117-39-5 |
| 97 | chaihu | Radix Bupleuri | MOL000354 | isorhamnetin | 49.6 | 0.31 | 480-19-3 |
| 98 | chaihu | Radix Bupleuri | MOL000422 | kaempferol | 41.88 | 0.24 | 520-18-3 |
| 99 | chaihu | Radix Bupleuri | MOL000449 | Stigmasterol | 43.83 | 0.76 | 83-48-7 |
| 100^*^ | chaihu | Radix Bupleuri | MOL000490 | petunidin | 30.05 | 0.31 | 1429-30-7 |
| 101 | chaihu | Radix Bupleuri | MOL001645 | Linoleyl acetate | 42.1 | 0.2 | 5999-95-1 |
| 102 | chaihu | Radix Bupleuri | MOL002776 | Baicalin | 40.12 | 0.75 | 21967-41-9 |
| 103 | chaihu | Radix Bupleuri | MOL004598 | 3,5,6,7-tetramethoxy-2-(3,4,5-trimethoxyphenyl)chromone | 31.97 | 0.59 | 17245-30-6 |
| 104 | chaihu | Radix Bupleuri | MOL004609 | Areapillin | 48.96 | 0.41 | 83162-82-7 |
| 105^*^ | chaihu | Radix Bupleuri | MOL004624 | Longikaurin A | 47.72 | 0.53 | 75207-67-9 |
| 106^*^ | chaihu | Radix Bupleuri | MOL004628 | Octalupine | 47.82 | 0.28 | 6809-89-8 |
| 107 | chaihu | Radix Bupleuri | MOL004644 | Sainfuran | 79.91 | 0.23 | 90664-32-7 |
| 108 | chaihu | Radix Bupleuri | MOL004648 | Troxerutin | 31.6 | 0.28 | 56764-99-9 |
| 109 | chaihu | Radix Bupleuri | MOL004653 | (+)-Anomalin | 46.06 | 0.66 | PNTWXEIQXBRCPS-JLTIQLCOSA-N |
| 110^*^ | chaihu | Radix Bupleuri | MOL004702 | saikosaponin c_qt | 30.5 | 0.63 | 20736-08-7 |
| 111 | chaihu | Radix Bupleuri | MOL004718 | α-spinasterol | 42.98 | 0.76 | 481-18-5 |
| 112 | chaihu | Radix Bupleuri | MOL013187 | Cubebin | 57.13 | 0.64 | 18423-69-3 |
| 113 | qinghao | Artemisia Annua L. | MOL000006 | luteolin | 36.16 | 0.25 | 491-70-3 |
| 114 | qinghao | Artemisia Annua L. | MOL000098 | quercetin | 46.43 | 0.28 | 117-39-5 |
| 115 | qinghao | Artemisia Annua L. | MOL000354 | isorhamnetin | 49.6 | 0.31 | 480-19-3 |
| 116 | qinghao | Artemisia Annua L. | MOL000359 | sitosterol | 36.91 | 0.75 | KZJWDPNRJALLNS-ZFVHJZABSA-N |
| 117 | qinghao | Artemisia Annua L. | MOL000422 | kaempferol | 41.88 | 0.24 | 520-18-3 |
| 118 | qinghao | Artemisia Annua L. | MOL000449 | Stigmasterol | 43.83 | 0.76 | 83-48-7 |
| 119 | qinghao | Artemisia Annua L. | MOL002235 | EUPATIN | 50.8 | 0.41 | 19587-65-6 |
| 120 | qinghao | Artemisia Annua L. | MOL004083 | Tamarixetin | 32.86 | 0.31 | 603-61-2 |
| 121 | qinghao | Artemisia Annua L. | MOL004112 | Patuletin | 53.11 | 0.34 | 519-96-0 |
| 122 | qinghao | Artemisia Annua L. | MOL004609 | Areapillin | 48.96 | 0.41 | 83162-82-7 |
| 123 | qinghao | Artemisia Annua L. | MOL005229 | Artemetin | 49.55 | 0.48 | 479-90-3 |
| 124 | qinghao | Artemisia Annua L. | MOL007274 | Skrofulein | 30.35 | 0.3 | 6601-62-3 |
| 125 | qinghao | Artemisia Annua L. | MOL007389 | artemisitene | 54.36 | 0.31 | 101020-89-7 |
| 126^*^ | qinghao | Artemisia Annua L. | MOL007400 | vicenin-2_qt | 45.84 | 0.21 | LYMBHOUDOYKJLM-UHFFFAOYSA-N |
| 127 | qinghao | Artemisia Annua L. | MOL007401 | Cirsiliol | 43.46 | 0.34 | 34334-69-5 |
| 128^*^ | qinghao | Artemisia Annua L. | MOL007404 | vitexin_qt | 52.18 | 0.21 | BGJVAMDCDFYLSR-UHFFFAOYSA-N |
| 129 | qinghao | Artemisia Annua L. | MOL007412 | DMQT | 42.6 | 0.37 | 5188-73-8 |
| 130 | qinghao | Artemisia Annua L. | MOL007415 | [(2S)-2-[[(2S)-2-(benzoylamino)-3-phenylpropanoyl]amino]-3-phenylpropyl] acetate | 58.02 | 0.52 | 56121-42-7 |
| 131^*^ | qinghao | Artemisia Annua L. | MOL007423 | 6,8-di-c-glucosylapigenin_qt | 59.85 | 0.21 | YTEIXAXHDMHUGP-UHFFFAOYSA-N |
| 132 | qinghao | Artemisia Annua L. | MOL007424 | artemisinin | 49.88 | 0.31 | 63968-64-9 |
| 133 | qinghao | Artemisia Annua L. | MOL007425 | dihydroartemisinin | 50.75 | 0.3 | 71939-50-9 |
| 134 | qinghao | Artemisia Annua L. | MOL007426 | deoxyartemisinin | 54.47 | 0.26 | 72826-63-2 |
| 135 | qianhu | Peucedani Radix | MOL000098 | quercetin | 46.43 | 0.28 | 117-39-5 |
| 136 | qianhu | Peucedani Radix | MOL000358 | beta-sitosterol | 36.91 | 0.75 | 83-46-5 |
| 137 | qianhu | Peucedani Radix | MOL000359 | sitosterol | 36.91 | 0.75 | KZJWDPNRJALLNS-ZFVHJZABSA-N |
| 138 | qianhu | Peucedani Radix | MOL001941 | Ammidin | 34.55 | 0.22 | 482-44-0 |
| 139 | qianhu | Peucedani Radix | MOL001942 | isoimperatorin | 45.46 | 0.23 | 482-45-1 |
| 140 | qianhu | Peucedani Radix | MOL002644 | Phellopterin | 40.19 | 0.28 | 2543-94-4 |
| 141 | qianhu | Peucedani Radix | MOL004653 | (+)-Anomalin | 46.06 | 0.66 | PNTWXEIQXBRCPS-JLTIQLCOSA-N |
| 142^*^ | qianhu | Peucedani Radix | MOL004792 | nodakenin | 57.12 | 0.69 | 495-31-8 |
| 143 | qianhu | Peucedani Radix | MOL005100 | 5,7-dihydroxy-2-(3-hydroxy-4-methoxyphenyl)chroman-4-one | 47.74 | 0.27 | AIONOLUJZLIMTK-CQSZACIVSA-N |
| 144 | qianhu | Peucedani Radix | MOL007154 | tanshinone iia | 49.89 | 0.4 | 568-72-9 |
| 145 | qianhu | Peucedani Radix | MOL013076 | (8S,9R)-9-hydroxy-8-(2-hydroxypropan-2-yl)-8,9-dihydrofuro[2,3-h]chromen-2-one | 37.3 | 0.2 | 53947-89-0 |
| 146 | qianhu | Peucedani Radix | MOL013077 | Decursin | 39.27 | 0.38 | 5928-25-6 |
| 147 | qianhu | Peucedani Radix | MOL013078 | praeruptorin E | 51.22 | 0.66 | UFUVJROSOIXJGR-MKKZQTCBSA-N |
| 148 | qianhu | Peucedani Radix | MOL013079 | dl-praeruptorin a | 46.46 | 0.53 | 73069-25-7 |
| 149 | qianhu | Peucedani Radix | MOL013081 | Decussine | 39.83 | 0.65 | 75375-52-9 |
| 150 | qianhu | Peucedani Radix | MOL013083 | Skimmin (8CI) | 38.35 | 0.32 | 93-39-0 |
| 151 | qianhu | Peucedani Radix | MOL013087 | Peucedanocoumarin II | 63.48 | 0.53 | LYUZYPKZQDYMEE-CCGSVPMNSA-N |
| 152 | qianhu | Peucedani Radix | MOL013093 | rubricauloside | 58.36 | 0.71 | 134018-84-1 |
| 153^*^ | qianhu | Peucedani Radix | MOL013094 | 8-[(2R)-2,3-dihydroxy-3-methyl-butyl]-5,7-dimethoxy-coumarin | 48.57 | 0.21 | 18196-00-4 |
| 154^*^ | qianhu | Peucedani Radix | MOL013095 | Sporidesmin | 58.31 | 0.76 | QTONANGUNATZOU-ICTVWZTPSA-N |
| 155 | qianhu | Peucedani Radix | MOL013098 | [(9R)-8,8-dimethyl-2-oxo-9,10-dihydropyrano[6,5-h]chromen-9-yl] (Z)-2-methylbut-2-enoate | 87.48 | 0.37 | 19427-82-8 |
| 156 | qianhu | Peucedani Radix | MOL013100 | (2S)-2-(1-hydroxy-1-methyl-ethyl)-9-[(2S,3R,4R,5S,6R)-3,4,5-trihydroxy-6-methylol-tetrahydropyran-2-yl]oxy-2,3-dihydrofuro[3,2-g]chromen-7-one | 45.33 | 0.73 | JWWFVRMFYKPZNE-CKFSJGLGSA-N |
| 157^*^ | qianhu | Peucedani Radix | MOL013101 | rutarin_qt | 70.1 | 0.2 | FVFQELHSZVFPDZ-VIFPVBQESA-N |
| 158 | qianhu | Peucedani Radix | MOL013103 | 532-16-1 | 46.57 | 0.44 | 532-16-1 |
| 159 | chuanbeimu | Fritiliariae Irrhosae Bulbus | MOL000358 | beta-sitosterol | 36.91 | 0.75 | 83-46-5 |
| 160 | chuanbeimu | Fritiliariae Irrhosae Bulbus | MOL000359 | sitosterol | 36.91 | 0.75 | KZJWDPNRJALLNS-ZFVHJZABSA-N |
| 161 | chuanbeimu | Fritiliariae Irrhosae Bulbus | MOL001749 | ZINC03860434 | 43.59 | 0.35 | BJQHLKABXJIVAM-WOJBJXKFSA-N |
| 162 | chuanbeimu | Fritiliariae Irrhosae Bulbus | MOL004440 | Peimisine | 57.4 | 0.81 | 19773-24-1 |
| 163 | chuanbeimu | Fritiliariae Irrhosae Bulbus | MOL009027 | Cyclopamine | 55.42 | 0.82 | 4449-51-8 |
| 164^*^ | chuanbeimu | Fritiliariae Irrhosae Bulbus | MOL009572 | Chuanbeinone | 41.07 | 0.71 | 103530-47-8 |
| 165^*^ | chuanbeimu | Fritiliariae Irrhosae Bulbus | MOL009579 | ent-(16S)-atisan-13,17-oxide | 47.74 | 0.43 | 84687-87-6 |
| 166 | chuanbeimu | Fritiliariae Irrhosae Bulbus | MOL009586 | isoverticine | 48.23 | 0.67 | 23496-43-7 |
| 167 | chuanbeimu | Fritiliariae Irrhosae Bulbus | MOL009588 | Korseveriline | 35.16 | 0.68 | 21851-05-8 |
| 168 | chuanbeimu | Fritiliariae Irrhosae Bulbus | MOL009589 | Korseverinine | 53.51 | 0.71 | 36506-64-6 |
| 169 | chuanbeimu | Fritiliariae Irrhosae Bulbus | MOL009593 | verticinone | 60.07 | 0.67 | IQDIERHFZVCNRZ-YUYPDVIUSA-N |
| 170^*^ | chuanbeimu | Fritiliariae Irrhosae Bulbus | MOL009596 | sinpemine A | 46.96 | 0.71 | 143120-47-2 |
| 171^*^ | chuanbeimu | Fritiliariae Irrhosae Bulbus | MOL009599 | songbeinone | 45.35 | 0.71 | 150133-32-7 |
| 172 | zhebeimu | Fritillariae Thunbrgii Bulbus | MOL000358 | beta-sitosterol | 36.91 | 0.75 | 83-46-5 |
| 173^*^ | zhebeimu | Fritillariae Thunbrgii Bulbus | MOL001004 | pelargonidin | 37.99 | 0.21 | SAURRTSFHXYOSN-UHFFFAOYSA-N |
| 174 | zhebeimu | Fritillariae Thunbrgii Bulbus | MOL004440 | Peimisine | 57.4 | 0.81 | 19773-24-1 |
| 175^*^ | zhebeimu | Fritillariae Thunbrgii Bulbus | MOL004443 | Zhebeiresinol | 58.72 | 0.19 | 151636-98-5 |
| 176 | zhebeimu | Fritillariae Thunbrgii Bulbus | MOL004444 | Ziebeimine | 64.25 | 0.7 | 130320-51-3 |
| 177 | zhebeimu | Fritillariae Thunbrgii Bulbus | MOL004446 | 6-Methoxyl-2-acetyl-3-methyl-1,4-naphthoquinone-8-O-beta-D-glucopyranoside | 33.31 | 0.57 | GVMOOQOCLYWVKF-BNCZOOBYSA-N |
| 178^*^ | zhebeimu | Fritillariae Thunbrgii Bulbus | MOL004450 | Chaksine | 65.63 | 0.66 | 486-53-3 |
| 179 | fuling | Poria Cocos(Schw.) Wolf. | MOL000273 | (2R)-2-[(3S,5R,10S,13R,14R,16R,17R)-3,16-dihydroxy-4,4,10,13,14-pentamethyl-2,3,5,6,12,15,16,17-octahydro-1H-cyclopenta[a]phenanthren-17-yl]-6-methylhept-5-enoic acid | 30.93 | 0.81 | XSLKAKROJKMHIT-WIUKAADNSA-N |
| 180 | fuling | Poria Cocos(Schw.) Wolf. | MOL000275 | trametenolic acid | 38.71 | 0.8 | 24160-36-9 |
| 181 | fuling | Poria Cocos(Schw.) Wolf. | MOL000276 | 7,9(11)-dehydropachymic acid | 35.11 | 0.81 | 77012-31-8 |
| 182 | fuling | Poria Cocos(Schw.) Wolf. | MOL000279 | Cerevisterol | 37.96 | 0.77 | 516-37-0 |
| 183 | fuling | Poria Cocos(Schw.) Wolf. | MOL000280 | (2R)-2-[(3S,5R,10S,13R,14R,16R,17R)-3,16-dihydroxy-4,4,10,13,14-pentamethyl-2,3,5,6,12,15,16,17-octahydro-1H-cyclopenta[a]phenanthren-17-yl]-5-isopropyl-hex-5-enoic acid | 31.07 | 0.82 | 6754-16-1 |
| 184 | fuling | Poria Cocos(Schw.) Wolf. | MOL000282 | ergosta-7,22E-dien-3beta-ol | 43.51 | 0.72 | QOXPZVASXWSKKU-UEIWAABPSA-N |
| 185 | fuling | Poria Cocos(Schw.) Wolf. | MOL000283 | Ergosterol peroxide | 40.36 | 0.81 | PIENIXCJUGJKPI-JYJMTLRPSA-N |
| 186 | fuling | Poria Cocos(Schw.) Wolf. | MOL000285 | (2R)-2-[(5R,10S,13R,14R,16R,17R)-16-hydroxy-3-keto-4,4,10,13,14-pentamethyl-1,2,5,6,12,15,16,17-octahydrocyclopenta[a]phenanthren-17-yl]-5-isopropyl-hex-5-enoic acid | 38.26 | 0.82 | 465-18-9 |
| 187 | fuling | Poria Cocos(Schw.) Wolf. | MOL000287 | 3beta-Hydroxy-24-methylene-8-lanostene-21-oic acid | 38.7 | 0.81 | UGMQOYZVOPASJF-OXUZYLMNSA-N |
| 188 | fuling | Poria Cocos(Schw.) Wolf. | MOL000289 | pachymic acid | 33.63 | 0.81 | 29070-92-6 |
| 189 | fuling | Poria Cocos(Schw.) Wolf. | MOL000290 | Poricoic acid A | 30.61 | 0.76 | 137551-38-3 |
| 190 | fuling | Poria Cocos(Schw.) Wolf. | MOL000291 | Poricoic acid B | 30.52 | 0.75 | 137551-39-4 |
| 191 | fuling | Poria Cocos(Schw.) Wolf. | MOL000292 | poricoic acid C | 38.15 | 0.75 | 151200-89-4 |
| 192 | fuling | Poria Cocos(Schw.) Wolf. | MOL000296 | hederagenin | 36.91 | 0.75 | KZJWDPNRJALLNS-CQXWNKEUSA-N |
| 193 | fuling | Poria Cocos(Schw.) Wolf. | MOL000300 | dehydroeburicoic acid | 44.17 | 0.83 | WZWOZRZYBSGARK-NUPWKHSHSA-N |
| 194 | wumei | Mume Fructus | MOL000098 | quercetin | 46.43 | 0.28 | 117-39-5 |
| 195 | wumei | Mume Fructus | MOL000358 | beta-sitosterol | 36.91 | 0.75 | 83-46-5 |
| 196 | wumei | Mume Fructus | MOL000422 | kaempferol | 41.88 | 0.24 | 520-18-3 |
| 197 | wumei | Mume Fructus | MOL000449 | Stigmasterol | 43.83 | 0.76 | 83-48-7 |
| 198 | wumei | Mume Fructus | MOL000953 | CLR | 37.87 | 0.68 | 57-88-5 |
| 199 | wumei | Mume Fructus | MOL001040 | (2R)-5,7-dihydroxy-2-(4-hydroxyphenyl)chroman-4-one | 42.36 | 0.21 | 480-41-1 |
| 200 | wumei | Mume Fructus | MOL005043 | campest-5-en-3beta-ol | 37.58 | 0.71 | 474-62-4 |
| 201 | wumei | Mume Fructus | MOL008601 | Methyl arachidonate | 46.9 | 0.23 | 2566-89-4 |
| 202 | xuanshen | Figwort Root | MOL000358 | beta-sitosterol | 36.91 | 0.75 | 83-46-5 |
| 203 | xuanshen | Figwort Root | MOL000359 | sitosterol | 36.91 | 0.75 | KZJWDPNRJALLNS-ZFVHJZABSA-N |
| 204 | xuanshen | Figwort Root | MOL001925 | paeoniflorin_qt | 68.18 | 0.4 | 23180-57-6 |
| 205 | xuanshen | Figwort Root | MOL002222 | sugiol | 36.11 | 0.28 | 511-05-7 |
| 206^*^ | xuanshen | Figwort Root | MOL007657 | scropolioside A_qt | 38.63 | 0.77 | JEVHQUKZENAMER-OUUQZIRNSA-N |
| 207^*^ | xuanshen | Figwort Root | MOL007658 | 14-deoxy-12(R)-sulfoandrographolide | 62.57 | 0.42 | LCOXPNVKZBRGJS-UQZPWQSVSA-N |
| 208 | xuanshen | Figwort Root | MOL007659 | scropolioside D | 36.62 | 0.4 | 148000-43-5 |
| 209^*^ | xuanshen | Figwort Root | MOL007660 | scropolioside D_qt | 33.17 | 0.82 | IDAIKLBZYZJEHE-LTUFEDGJSA-N |
| 210^*^ | xuanshen | Figwort Root | MOL007662 | harpagoside_qt | 122.87 | 0.32 | IQLOULSXIQEXGV-FMGMVOTKSA-N |
| 211 | huangqi | Hedysarum Multijugum Maxim. | MOL000033 | (3S,8S,9S,10R,13R,14S,17R)-10,13-dimethyl-17-[(2R,5S)-5-propan-2-yloctan-2-yl]-2,3,4,7,8,9,11,12,14,15,16,17-dodecahydro-1H-cyclopenta[a]phenanthren-3-ol | 36.23 | 0.78 | 64997-52-0 |
| 212 | huangqi | Hedysarum Multijugum Maxim. | MOL000098 | quercetin | 46.43 | 0.28 | 117-39-5 |
| 213 | huangqi | Hedysarum Multijugum Maxim. | MOL000211 | Mairin | 55.38 | 0.78 | 472-15-1 |
| 214 | huangqi | Hedysarum Multijugum Maxim. | MOL000239 | Jaranol | 50.83 | 0.29 | 3301-49-3 |
| 215 | huangqi | Hedysarum Multijugum Maxim. | MOL000296 | hederagenin | 36.91 | 0.75 | KZJWDPNRJALLNS-CQXWNKEUSA-N |
| 216 | huangqi | Hedysarum Multijugum Maxim. | MOL000354 | isorhamnetin | 49.6 | 0.31 | 480-19-3 |
| 217 | huangqi | Hedysarum Multijugum Maxim. | MOL000371 | 3,9-di-O-methylnissolin | 53.74 | 0.48 | RFFNFQZKHNKOPO-BBRMVZONSA-N |
| 218^*^ | huangqi | Hedysarum Multijugum Maxim. | MOL000374 | 5'-hydroxyiso-muronulatol-2',5'-di-O-glucoside | 41.72 | 0.69 | SRVGYVIWVOOXQO-FQRJZKGRSA-N |
| 219 | huangqi | Hedysarum Multijugum Maxim. | MOL000378 | 7-O-methylisomucronulatol | 74.69 | 0.3 | BLHQCBJSTMDZQA-LBPRGKRZSA-N |
| 220 | huangqi | Hedysarum Multijugum Maxim. | MOL000379 | 9,10-dimethoxypterocarpan-3-O-β-D-glucoside | 36.74 | 0.92 | 94367-42-7 |
| 221 | huangqi | Hedysarum Multijugum Maxim. | MOL000380 | (6aR,11aR)-9,10-dimethoxy-6a,11a-dihydro-6H-benzofurano[3,2-c]chromen-3-ol | 64.26 | 0.42 | 73340-41-7 |
| 222 | huangqi | Hedysarum Multijugum Maxim. | MOL000387 | Bifendate | 31.1 | 0.67 | 73536-69-3 |
| 223 | huangqi | Hedysarum Multijugum Maxim. | MOL000392 | formononetin | 69.67 | 0.21 | 485-72-3 |
| 224 | huangqi | Hedysarum Multijugum Maxim. | MOL000398 | isoflavanone | 109.99 | 0.3 | JNSVNRWHSLLCBG-LLVKDONJSA-N |
| 225 | huangqi | Hedysarum Multijugum Maxim. | MOL000417 | Calycosin | 47.75 | 0.24 | 20575-57-9 |
| 226 | huangqi | Hedysarum Multijugum Maxim. | MOL000422 | kaempferol | 41.88 | 0.24 | 520-18-3 |
| 227 | huangqi | Hedysarum Multijugum Maxim. | MOL000433 | FA | 68.96 | 0.71 | OVBPIULPVIDEAO-LBPRGKRZSA-N |
| 228 | huangqi | Hedysarum Multijugum Maxim. | MOL000438 | (3R)-3-(2-hydroxy-3,4-dimethoxyphenyl)chroman-7-ol | 67.67 | 0.26 | 64474-51-7 |
| 229 | huangqi | Hedysarum Multijugum Maxim. | MOL000439 | isomucronulatol-7,2'-di-O-glucosiole | 49.28 | 0.62 | NHOPAJCVMDIGBN-MEPKZADGSA-N |
| 230 | huangqi | Hedysarum Multijugum Maxim. | MOL000442 | 1,7-Dihydroxy-3,9-dimethoxy pterocarpene | 39.05 | 0.48 | RVGZSUMTFIEORY-UHFFFAOYSA-N |
| 231 | taizishen | Pseudostellariae Radix | MOL000006 | luteolin | 36.16 | 0.25 | 491-70-3 |
| 232 | taizishen | Pseudostellariae Radix | MOL000358 | beta-sitosterol | 36.91 | 0.75 | 83-46-5 |
| 233 | taizishen | Pseudostellariae Radix | MOL001506 | Supraene | 33.55 | 0.42 | 111-02-4 |
| 234 | taizishen | Pseudostellariae Radix | MOL001689 | acacetin | 34.97 | 0.24 | 480-44-4 |
| 235 | taizishen | Pseudostellariae Radix | MOL001790 | Linarin | 39.84 | 0.71 | 480-36-4 |
| 236 | taizishen | Pseudostellariae Radix | MOL002464 | 1-Monolinolein | 37.18 | 0.3 | WECGLUPZRHILCT-GSNKCQISSA-N |
| 237 | taizishen | Pseudostellariae Radix | MOL006554 | Taraxerol | 38.4 | 0.77 | 127-22-0 |
| 238 | taizishen | Pseudostellariae Radix | MOL006756 | Schottenol | 37.42 | 0.75 | 521-03-9 |
